# 

**Published:** 1877-04

**Authors:** 


SURG GENLS OFFS

Vol. XIII. APRIL, 1877. No. 1.

### THE BISTOURY:

### A QUARTERLY JOURNAL,

*Devoted to the Exposition of Charlatanism in Medicine and the Education of the People upon Medical Subjects !*

Thad S. Up de Graff, M. D., Editor.

*Terms of Subscription, Fifty-five Cents per Annum, in Advance.*

ELMIRA, N. Y.

PRINTED FOR THE PROPRIETOR, AT THE DAILY ADVERTISER JOB PRINTING HOUSE.

